# Ocean Genomes: reference genome resources for marine vertebrates

**DOI:** 10.1038/s44185-025-00109-2

**Published:** 2025-10-01

**Authors:** Lara Parata, Emma de Jong, Richard J. Edwards, Philipp E. Bayer, Liam Anstiss, Stephen R. Burnell, Adrianne Doran, Priscila Goncalves, Lauren Huet, Glenn I. Moore, Tyler E. Peirce, OceanOmics Centre, Lara Parata, Richard J. Edwards, Philipp E. Bayer, Liam Anstiss, Stephen R. Burnell, Adrianne Doran, Priscila Goncalves, Lauren Huet, Marcelle E. Ayad, Adam J. Bennett, Emma de Jong, Anna Depiazzi, Ibrahim Faseeh, Matthew W. Fraser, Sang Huynh, Anya Kardailsky, Laura Missen, Georgia M. Nester, Tyler E. Peirce, Eric J. Raes, Ebony M. Thorpe, Shannon Corrigan, OceanOmics Division, Philipp E. Bayer, Stephen R. Burnell, Priscila Goncalves, Marcelle E. Ayad, Adam J. Bennett, Matthew W. Fraser, Anya Kardailsky, Georgia M. Nester, Eric J. Raes, Ebony M. Thorpe, Michael Bunce, Madalyn K. Cooper, Jessica R. Pearce, Sebastian Rauschert, Julie C. Robidart, Shannon Corrigan, Shannon Corrigan

**Affiliations:** 1 Minderoo OceanOmics Centre at UWA, Oceans Institute, University of Western Australia, Perth, WA Australia; 2 Minderoo Foundation, Perth, WA Australia; 3 Collections and Research, Western Australian Museum, Welshpool, WA Australia; 4 School of Biological Sciences, University of Western Australia, Perth, WA Australia

**Keywords:** Ecology, Evolution, Genetics, Genome, Genomics, Research data

## Abstract

We present Ocean Genomes, a program dedicated to producing reference genome resources to facilitate improved monitoring approaches and management outcomes for marine vertebrate biodiversity. Ocean Genomes will generate high-quality reference genomes of representatives of all marine vertebrate families and additional high-conservation-value species. Draft-quality genomes may be produced for a more comprehensive sampling of species. We include case studies of *Enoplosus armatus*, Old Wife and *Pempheris klunzingeri*, Rough Bullseye.

## Introduction

Reference genomes are a foundational resource in contemporary biology, underpinning breakthroughs across various scientific domains such as medicine, agriculture, biodiversity, ecology, conservation and evolution. Indeed, increasing demand for these data and associated technological advancements in DNA sequencing and computing has resulted in a new era for reference genome generation for organisms across the Tree of Life^1–3^. For example, global initiatives such as the Earth BioGenome Project (EBP)^4^ are underway, aiming to compile reference genomes for all eukaryotic species. While impressive, the task is vast, and so moonshot initiatives such as this operate as a global collaboration of affiliated projects, each targeting portions of regional, ecosystem or taxonomic diversity that align with their respective project goals. For example, since its launch in 2018, the EBP has grown to include 58 affiliated projects (https://www.earthbiogenome.org/, accessed 18/10/2024) that typically have operational focal points, such as biogeographic region (e.g. Darwin Tree of Life^5^, African BioGenome Project^6^, European Reference Genome Atlas)^7^, ecosystems (e.g. PhyloAlps)^8^ or taxa of interest (e.g. The Vertebrate Genomes Project (VGP)^9^, 10,000 Bird Genomes (B10K)^10^, 10,000 Plant Genomes (10KP)^11^ and Oz Mammals Genomics)^12^.

**Fig. 1:**
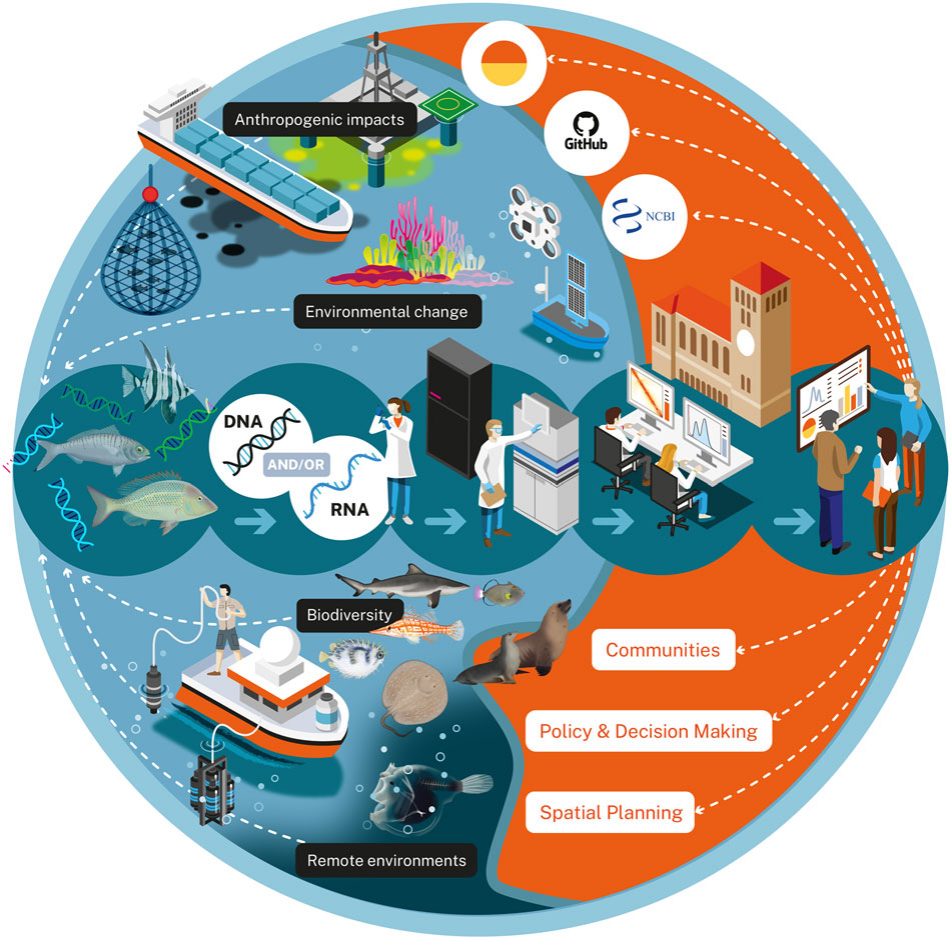
OceanOmics overview. A primary goal of OceanOmics is to develop environmental DNA (eDNA) as a cost-effective, scalable biomonitoring tool. Key activities include producing eDNA biodiversity and high-quality reference genome datasets at scale, making these data available and translating information for communities and to influence policy and decision-making frameworks.

In this paper, we describe Ocean Genomes, one such EBP-affiliated project. Ocean Genomes aims to generate high-quality reference genomic resources that can support broader programme goals to develop environmental DNA (eDNA) as a scalable biodiversity sampling solution. Like all EBP-affiliated projects, it is anticipated that Ocean Genomes resources will also serve as foundational resources supporting multidisciplinary scientific research and outcomes. Ocean Genomes is enabled by Minderoo Foundation OceanOmics (Perth, Australia) and the University of Western Australia (Perth, Australia) via the Minderoo OceanOmics Centre at UWA (Fig. 1). Below, we describe aspects of Ocean Genomes strategic focus and approach, recognising that generation and impactful use of high-quality reference genomic resources requires a coordinated and collaborative effort among many stakeholders.

A core aim of the Minderoo Foundation OceanOmics programme is to develop eDNA as an enabling technology supporting routine ocean-scale biodiversity discovery and monitoring. Marine vertebrates native to Australian waters and the Indo-Pacific region are the primary focus of OceanOmics and thus Ocean Genomes, strategically aiming to fill gaps across existing environmental metagenomics initiatives (e.g. KAUST Metagenomic Analysis Platform^13^, Ocean Genome Atlas Project^14^, *Tara* Oceans)^15^ and in available reference sequence (RefSeq) databases. At the time of Ocean Genomes conception, just 3.5% of marine vertebrate species had a reference-quality whole genome sequence available in public repositories. Those that were available typically represented Northern Hemisphere diversity^16^. Located with geographic proximity to the Indian Ocean on the western coastline of Australia, a key role of Ocean Genomes is to contribute openly accessible reference genomic resources for marine vertebrate diversity that is currently underrepresented in public repositories. Moreover, the focal region encompasses 8.9 million square kilometres^17^ of crucial habitat for ~5500 marine vertebrate species^18^, more than 300 of which are categorised as Threatened or Near Threatened according to the International Union for Conservation of Nature Red List of Threatened Species^19^. Applying this taxonomic and regional focus will allow priority generation of reference resources that can support positive conservation science and management outcomes.

Ocean Genomes strives to represent the biological diversity of marine vertebrates with reference genome resources that meet the quality standards (more below) of the EBP^4,20,21^ and affiliated VGP^9^. We follow best practice guidance of the EBP in our approaches to identifying sequencing priorities and sampling specimens^21^. Coordinating efforts with global genome sequencing consortia, Ocean Genomes will initially target a representative species for each of the ~495 marine vertebrate families, expanding to represent greater species diversity with high-quality reference genome resources over time. Representative species selection prioritises those that are of perceived high conservation value, such as threatened, commercially important, keystone, indicator, or regionally endemic species or taxa that are significant to Indigenous Peoples and Local Communities. A representative species may also be prioritised for high-quality reference genome generation if the resource will benefit Australian and regional scientific, conservation and management outcomes.

Additional to the primary goal, draft quality reference genome assemblies or population-level whole genome resequencing datasets may be produced to facilitate more comprehensive representation of Australian and regionally important marine vertebrate diversity in public sequence repositories, recognising that such resources are highly enabling for many applications including those aligned with our specific interests to facilitate eDNA based biomonitoring and enhance understanding of the taxonomy, biology and ecology of marine vertebrates to support their conservation and management (Fig. 1).

To ensure the production of authoritative, high-quality reference genomes, Ocean Genomes aligns with best practices for cataloguing biological diversity. In addition to the criteria above, representative species are selected based on their taxonomy being relatively stable with publicly registered nomenclature that is traceable in faunal databases (e.g. National Center for Biotechnology Information (NCBI) Taxonomy Database^22^; Australian Faunal Directory^23^; World Register of Marine Species (WoRMS)^24^; Eschmeyer’s Catalog of Fishes^25^). All assemblies are accompanied by comprehensive metadata, including geolocation, environmental and collection method information. Wherever possible, high-quality images of the specimen in fresh colouration and voucher samples and specimens are also collected. We endeavour to work with regional experts, primarily collections scientists, so that specimen vouchers can be expertly identified, deposited in a registered collection close to the place of provenance, curated, and maintained to allow initial and repeat (in scenarios of taxonomic flux) verification of the nominal species identity that is assigned to the reference genome assembly. In the case of smaller organisms where specimens will likely be exhausted during processing, additional individuals are sampled from the same time and place, and co-identity is verified via photographic vouchers and barcode sequence matches (see more on our approach to molecular validation in case studies below).

Prior to prioritising a representative species for sequencing, we consult genome-relevant metadata, project plans and statuses of similarly aligned efforts (e.g. EBP affiliated projects, 10,000 Fish Genomes Project)^26^ via publicly available indexes (e.g. Genomes on a Tree (GoaT)^27^ and Australian Reference Genome Atlas)^28^ and repositories (e.g. Australasian Genomes^29^ and The RefSeq^30^ collection of the NCBI)^31^ to avoid unnecessary depletion of resources and/or duplication of effort.

Producing high-quality data types to meet the EBP and VGP quality standards typically requires fresh collection of tissues, from which high-molecular-weight DNA can be extracted^9,20^. This often requires sampling from live or freshly euthanised individuals so that samples can be immediately flash-frozen in liquid nitrogen, remaining cryopreserved until processing for DNA extraction and sequencing. The collection of fresh samples reduces the risk of DNA degradation from cellular enzymes, ice crystal formation or chemical preservation, improving high-molecular-weight DNA yields^32^. The need to collect fresh specimens and samples can limit global genome sequencing efforts^20^, especially when targeting rare or threatened species^4,20^. Sampling remote marine locations and environments, including finding, transporting and handling liquid nitrogen and potentially large animals under marine field work conditions, are particularly challenging. These are some reasons that marine vertebrate species, particularly those wide-ranging and elusive species, are underrepresented by high-quality reference genome assemblies. The critical importance of multistakeholder collaboration extends from setting sequencing priorities to identifying and executing upon achievable opportunities for sampling. Ocean Genomes endeavours to collaborate, prioritise, sample, sequence and share data (see more in data sharing and availability), in the place of specimen provenance, operating in accordance with local conventions and laws to ensure ethical and legal sample collection and equitable access to benefits. For bony fishes (which constitute most of our target species), we aim to collect more than 100 mg from multiple tissue types, balancing speed to preservation with maintaining the external morphological integrity of the voucher specimen. Typically, this means removing tissue from the right-side rear gills and excising muscle, liver and heart via a small incision in the belly. Samples are flash frozen in dry tubes and RNA-later as sub-sampled pieces to allow independent thawing at the time of preparation for long-read, high-throughput chromatin conformation capture (Hi-C), and transcriptome sequencing (avoiding unnecessary freeze-thaw cycles. A blood draw (at least 500 µL preserved 1:10 in chilled absolute Ethanol and 1:5 in RNA-later) and minimally invasive muscle biopsy are preferred for particularly vulnerable species such as cartilaginous fishes (chimaeras, sharks, skates, rays) and marine mammals. In these cases, samples are only taken by experienced handlers and in accordance with ethics and permits approvals. Species identity is vouchered by a photo image.

Ocean Genomes aims to produce high-quality, near error-free, near-complete, chromosome-level, annotated reference genome assemblies for a representative species of all marine vertebrate families, plus additional representatives of high-conservation value groups. To achieve this, we are combining single-molecule long-read data for contig building (PacBio HiFi; Menlo Park, California), long-range data from high-throughput chromosome conformation capture (Hi-C; Dovetail® Omni-C™ and Dovetail® LinkPrep™, Cantata Bio, Scotts Valley, California) sequenced with short-reads (Illumina, San Diego, California) for scaffolding, and transcriptomic data (Illumina® stranded mRNA prep and PacBio Kinnex full-length RNA) for annotation. We are striving to generate, assemble and annotate phased chromosome-level genomes with quality metrics that satisfy the EBP version 6.0—September 2024 6.C.Q40^33^ and VGP 7.c.P6.Q50.C95 standards^9^, including contiguity (NG50 > 10 Mb), base accuracy (QV > 50), functional completeness (assembled genes >95% complete) and chromosome assembly (>95% assigned to chromosomes). Where possible, we sequence DNA derived from the heterogametic sex to allow all sex chromosomes to be represented by the assembly.

When fresh tissues and suitably high molecular weight (HMW) DNA are unable to be collected to support high-quality reference genome sequencing and assembly, Ocean Genomes will instead generate a draft-quality genome assembly that is based on ~50× coverage of short-read data (Illumina). While these assemblies are characterised by lower contiguity, higher base ambiguity and a smaller percentage of sequences assembled onto chromosomes^34^, they are nevertheless subject to stringent internal quality control, including molecular validation of nominal specimen identification wherever possible and promote the inclusion of a wider range of species and marine vertebrate diversity among Ocean Genomes resources.

**Fig. 2:**
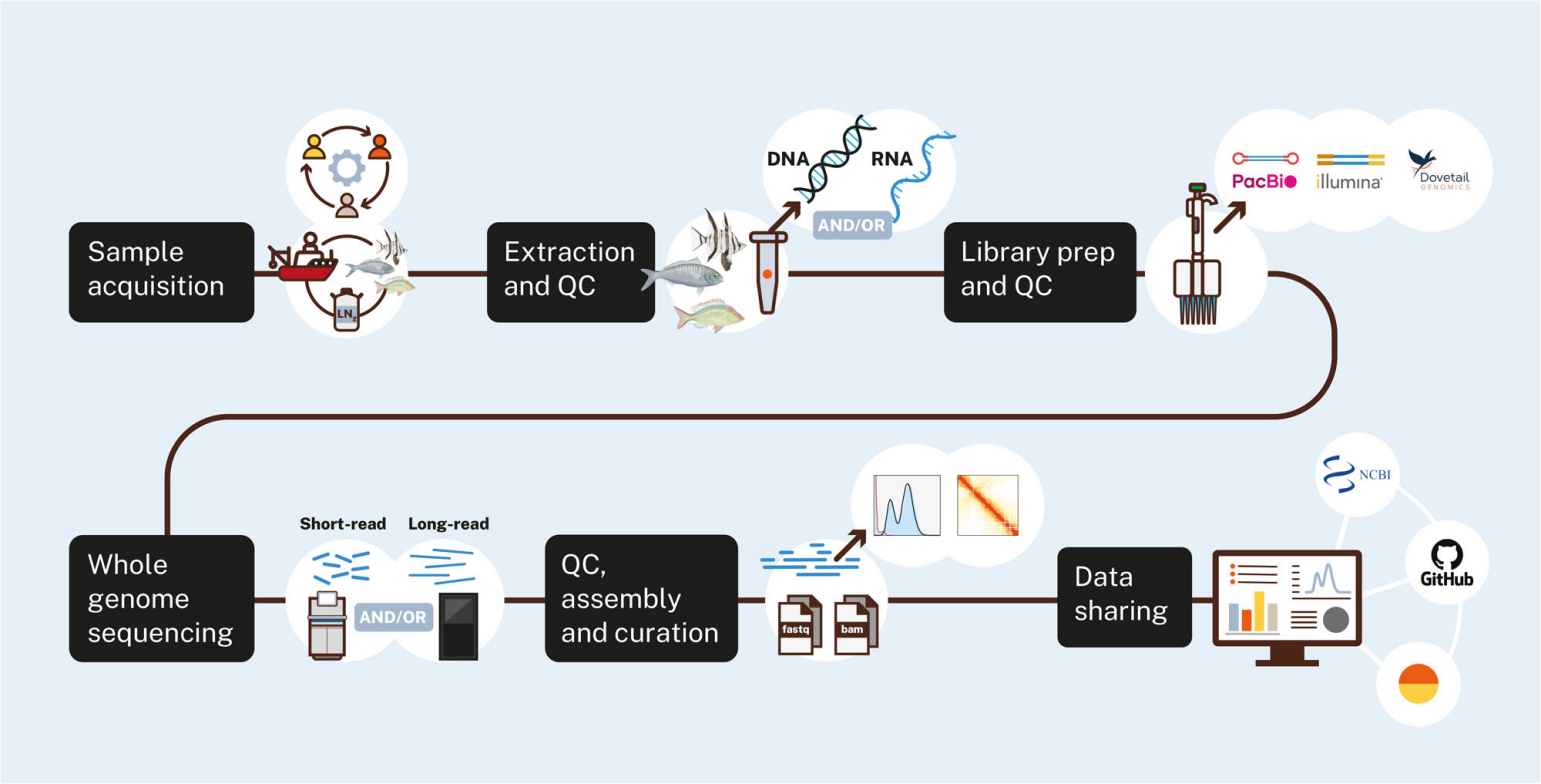
An overview of Ocean Genomes workflows. Step 1—Sample acquisition, including identifying target species, engagement with relevant stakeholders to identify opportunities and strategy for sampling, voucher specimen and sample collection, expert identification, sample, specimen and metadata accessioning. Step 2—DNA and/or RNA extraction from tissue samples & quality assessment. Step 3—PacBio HiFi, Hi-C and transcriptome (Illumina RNA-Seq and PacBio Iso-Seq) library preparation & quality assessment. Step 4—Whole Genome Sequencing via short (Illumina) or long-read (PacBio HiFi) technologies & quality assessment. Step 5—Draft or reference quality genome assembly, quality assessment and manual curation. Step 6—Sequencing data and associated assemblies are openly accessible via custom and established public sequence repositories (Table 1).

Open research data promotes equitable access to benefits and accelerates scientific progress and transparency while minimising duplication of effort and resource allocation. Ocean Genomes endorses principles of open access science, research and data outputs, adopting FAIR guiding principles for scientific data management and stewardship^35^. All Ocean Genomes sequencing data and genome assemblies will be openly accessible in the public domain and available for use under a Creative Commons Attributions license CC BY 4.0. A customised Minderoo OceanOmics dashboard provides regular updates to the community regarding collaborations, specimens acquired and prioritised for sequencing, the type of reference genome being produced and the progress of a sample from collection through to final assembly and data sharing. The dashboard connects users to open repositories (NCBI and Amazon Web Services) where data and supporting resources are available for download (Fig. 2). In future iterations of the dashboard, we intend to share standardised genome notes that promote the reuse of the data, and invite collaboration and disclosure of cultural authority and traditional knowledge interests of indigenous peoples and local communities, for example by incorporating biocultural, traditional knowledge and engagement notices (e.g. via institutional implementations of the CARE principles, or via the Local Contexts Notices system https://localcontexts.org/.)^36^

Ocean Genomes sequencing data and genome assemblies are also accessible directly via NCBI under BioProject number PRJNA1046164 and the affiliated Sequence Read Archive (SRA) or GenBank records. Progress toward high-quality reference genome production is also reported via GoaT^27^ as part of coordinated efforts across the EBP.

High-quality reference genome assembly and quality assessment follow VGP workflows^9^. Draft genome assembly and quality assessment follow custom pipelines. All associated code is shared via GitHub. Links to publicly accessible Ocean Genomes resources are provided in Table 1.

**Table 1:** Publicly accessible Ocean Genomes resources

Resource	Type	Link
OceanOmics Dashboard	Progress tracking and data	https://edna.minderoo.org/
Ocean Genomes NCBI BioProject	Data	https://www.ncbi.nlm.nih.gov/bioproject/1046164
Minderoo Foundation GitHub	Analyses, pipelines and code	https://github.com/MinderooFoundationhttps://github.com/MinderooFoundation/OceanOmics-OceanGenomes-ref-genomeshttps://github.com/MinderooFoundation/OceanOmics-OceanGenomes-draft-genomes
Genomes on a Tree (GoaT)	Progress tracking	https://goat.genomehubs.org/projects/OG

## Proof of concept: high-quality reference genome assemblies of *Enoplosus armatus* (Shaw 1790) and *Pempheris klunzingeri* McCulloch 1911

**Fig. 3:**
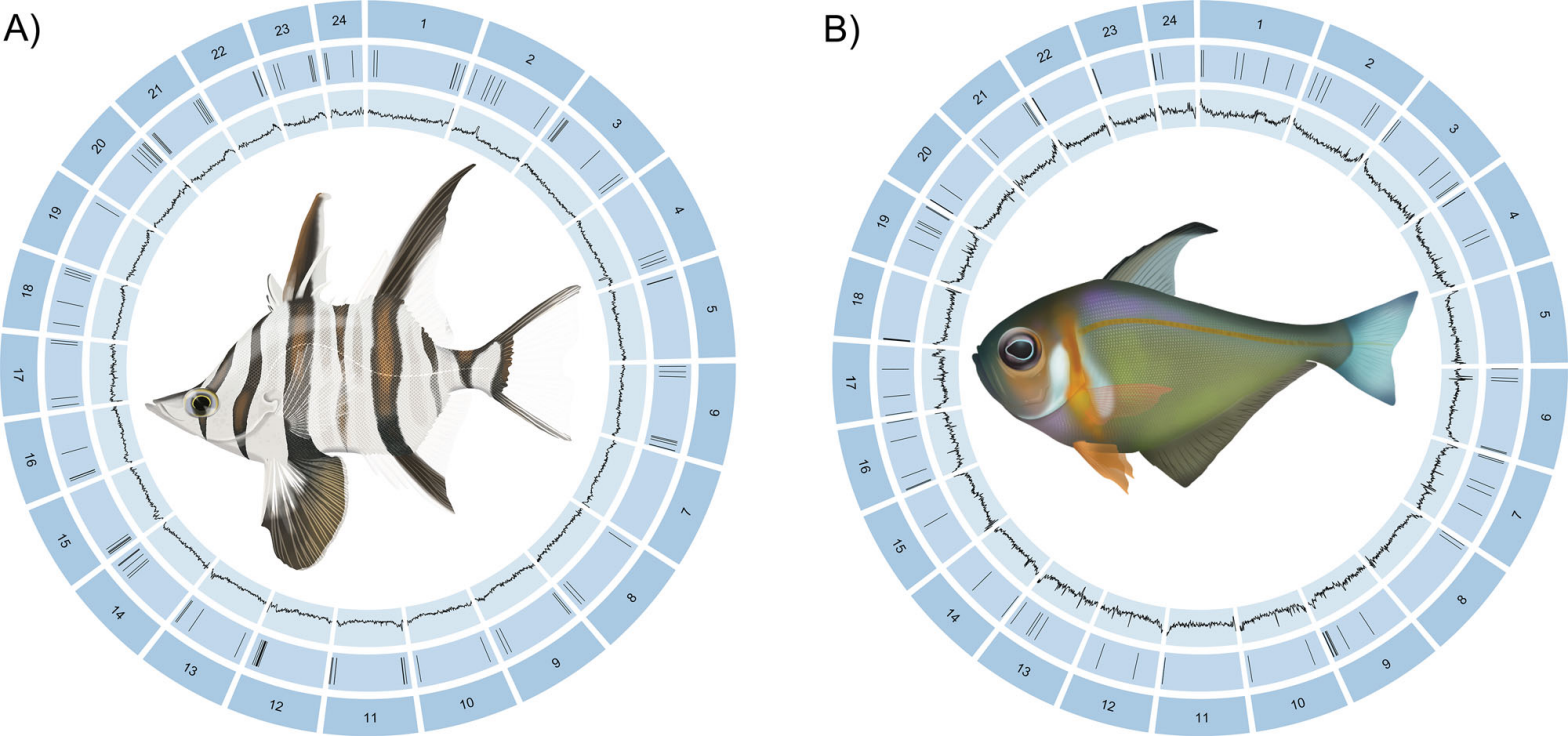
Genome attributes of *Enoplosus armatus* and *Pempheris klunzingeri*. Features of phased haplotype 1 chromosomes **A** *E. armatus* (fEnoArm2) and **B** *P. klunzingeri* (fPemKlu1) reference genomes. Concentric tracks from the outside inward represent chromosomes (numbered by length), gaps (gaps of unknown length appear as 100 bp in the assembly) and GC content calculated using BEDTools version 2.31.1^67^ using a sliding window of 10,000 bp. Visualisation created using the R package circlize version 0.4.16^68^.

To share our methods and demonstrate the types of resources that will be produced by Ocean Genomes, we present high-quality, near error-free and gapless, chromosome-level, haplotype-phased and curated, reference genome assemblies for two marine fishes: *E. armatus* (Shaw 1790), Old Wife, (family: Enoplosidae); and *P. klunzingeri* McCulloch 1911, Rough Bullseye, (family: Pempheridae) (Fig. 3). Both *E. armatus* and *P. klunzingeri* are Australian endemics. The assemblies described here constitute the first high-quality reference genomes representing families Enoplosidae and Pempheridae.

*E. armatus* occurs across sub-tropical to temperate Australian waters, where climate-driven environmental changes are affecting their population numbers and distribution^37^. *E. armatus* is the only extant species of the family Enoplosidae, which has an uncertain phylogenetic position^38^ based on conflicting signals from mitogenome data^39,40^ and nuclear markers^41^. It is anticipated that this reference genome may represent a resource for resolving phylogenetic uncertainty as well as understanding the molecular basis of local adaptations and traits undergoing selection, informing conservation efforts for the species^2^.

*P. klunzingeri* is endemic to the waters of the southwest coast of Australia and is facing similar threats from climate change as *E. armatus*. Prior to this study, there were no genetic data available in public sequence repositories for the species. It is anticipated that representing this diversity in refSeq repositories may improve the resolution of eDNA biomonitoring tools and increase the understanding of interesting adaptive traits present in this group of fishes, such as their nocturnal behaviour^42^ or the evolution of their bioluminescent organ^43,44^.

### Specimen collection

In April 2023, researchers from Minderoo Foundation OceanOmics Division and Western Australian Museum (WAM) conducted a joint campaign to characterise marine vertebrate diversity along the coastline of southwestern Australia, combining eDNA sampling along with in-water surveys and specimen collection. Adult specimens of *E. armatus* and *P. klunzingeri* were collected by GIM (WAM) on SCUBA with a hand spear near Middle Island (*E. armatus*) and New Year Island (*P. klunzingeri*) of Wudjari Nyungar Sea Country, Recherche Archipelago, Western Australia. The specimens were humanely euthanised following expert taxonomic identification. Specimens were pinned and imaged in fresh colouration by GIM (WAM). Samples of liver, gills and muscle tissue were then aseptically dissected from the *E. armatus* specimen and flash-frozen in a liquid nitrogen dewar. Due to its small size, the whole *P. klunzingeri* specimen was flash-frozen in liquid nitrogen. Flash-frozen samples were transported to Minderoo OceanOmics Centre at UWA (Perth, Australia), where they remained at −80 °C until the time of laboratory processing. Voucher specimens were preserved in formalin in the field by GIM (WAM) and subsequently accessioned into the WAM as follows: *E. armatus*—WAM P.35492-002, and *P. klunzingeri*—WAM P.35483-003.

Research activities were conducted under Access to Biological Resources in a Commonwealth Area for Non-Commercial Purposes permit numbers AU-COM2020-498 and AU-COM2020-499 and Australian Marine Park Activity Permit numbers PA2021-00009-4 and PA2020-00048-1. Specimens were collected under Western Australian Government Department of Biodiversity Conservation and Attractions fauna taking (scientific or other purposes) licence number FO25000006-24 and Department of Primary Industries WA Fisheries Fish Resources Management Act 1994 exemption number 250966222. At the time of sampling in Western Australia, the Animal Welfare Act 2002 did not require WAM to obtain animal ethics committee approval of care and use of fishes. Nonetheless, sampling was undertaken in strict adherence to the state government Department of Biodiversity, Conservation and Attractions and WAM standard operating procedures for the safe and humane handling, use and care of marine fauna for research purposes.

### DNA/RNA extractions, library preparations and sequencing

Extraction, library preparations and sequencing followed the protocols described in Parata et al.^45^, and are summarised herein. HMW genomic DNA was extracted from approximately 25 mg of gill tissue for both *E. armatus* and *P. klunzingeri*. Tissues were homogenised and pelleted as per the PacBio Nanobind tissue kit (PacBio, CA, USA) protocol using the TissueRuptor II (QIAGEN, Hilden, Germany). Cell lysis and DNA isolation were performed following the PacBio “Extracting HMW DNA from skeletal muscle using Nanobind” procedure (102-579-200, Dec 2022). The quantity and fragment length distribution of extracted gDNA were determined using a Qubit 3 Fluorometer with the Qubit dsDNA Broad-Range Assay Kit (Thermo Fisher Scientific, MA, USA), a NanoDrop One (Thermo Fisher Scientific, MA, USA) and a Femto Pulse with the Genomic DNA 165 kb kit (Agilent, CA, USA). PacBio HiFi SMRTbell® libraries were prepared using the PacBio SMRTbell® prep kit 3.0 (PacBio, CA, USA) according to manufacturer’s instructions. The SMRTbell-polymerase complexes were each sequenced across two SMRT Cells (8 M) on a PacBio Sequel IIe (targeting ~40× coverage of the genome) with movie times of 30 h, producing data outputs and average read lengths as described in Table 2.

**Table 2:** Raw sequencing data for *Enoplosus armatus* (fEnoArm2) *and Pempheris klunzingeri* (fPemKlu1)

	HiFi			Hi-C		NCBI BioProject
	Average read length (bp)	Data (Gb)	Coverage (×)	Data (Gb)	Coverage (×)	
*Enoplosus armatus* (fEnoArm2)	10,500	54	103	33	62	PRJNA1074348
*Pempheris klunzingeri* (fPemKlu1)	12,450	61	104	40	68	PRJNA1079283

Frozen liver (*E. armatus*) and gill (*P. klunzingeri*) tissue were ground in liquid nitrogen to facilitate the construction of chromatin conformation capture proximity ligation (Hi-C)^46^ libraries using the Dovetail Omni-C proximity Ligation Assay kit, with the Dovetail Omni-C Module and Dovetail Library Module for Illumina kits (Cantata Bio, CA, USA), as per the manufacturer's protocols. The Omni-C method of acquiring Hi-C data was chosen as it uses a sequence-independent endonuclease, rather than restriction enzymes, to digest chromatin, providing more uniform sequencing coverage across the genome^47,48^. Library complexity was assessed by shallow sequencing the Hi-C libraries on an Illumina iSeq 100 system using a 2 × 150 bp paired-end run. Deep sequencing (targeting ~60× coverage of the genome) was then carried out on an Illumina NextSeq 2000 platform with a 2 × 150 bp paired-end run configuration to generate chromosome conformation data (Table 2).

Total RNA was extracted separately from gill and muscle tissue for both *E. armatus* and *P. klunzingeri* using the Monarch® Total RNA Miniprep Kit (New England Biolabs, MA, USA) following the manufacturer's protocol. Extracted RNA was then quantified and quality checked using NanoDrop One (Thermo Fisher Scientific, MA, USA), a Qubit 3 Fluorometer with the Qubit HS RNA Kit (Thermo Fisher Scientific, MA, USA) and TapeStation 4150 system with High Sensitivity RNA ScreenTape (Agilent, CA, USA). Extracts were subsequently concentrated and/or cleaned using the Monarch® RNA Cleanup Kit (New England Biolabs, MA, USA). RNA-Seq libraries were constructed using Illumina Stranded mRNA Prep and sequenced on an Illumina NovaSeq 6000 using a 2 × 150 bp paired-end run configuration (targeting 50 million paired-end reads per tissue). The resulting reads were quality control checked with FastQC (v0.11.9)^49^ and fastp (v0.23.2)^50^ to remove adaptor contamination, ready for downstream use. For each species, a further 300 ng of total RNA was extracted from gill and muscle tissues as above and converted to full-length cDNA using the Iso-Seq® Express 2.0 Kit (PacBio, CA, USA), following the manufacturer's protocol. The resulting cDNA was then processed with the Kinnex™ Full-Length RNA Kit (PacBio, CA, USA) to generate concatenated full-length RNA libraries, which were sequenced with long reads on a PacBio Revio™ System (targeting approximately 5 million concatenated reads per library). The resulting HiFi reads were processed using the Iso-Seq workflow (v4.3.0)^51^ to remove cDNA primers, polyA tails and artificial concatemers, generating demultiplexed full-length non-chimeric reads, followed by clustering to generate consensus high-quality isoforms.

### Genome assembly, curation, quality assessment and annotation

Near error-free and gapless, chromosome-level, haplotype-phased and curated genome assemblies for *E. armatus* and *P. klunzingeri* were generated using PacBio HiFi long-read data and Illumina-sequenced Hi-C data following established workflows^52^. Briefly, raw HiFi reads were quality control checked using HiFiAdapterFilt (v2.0)^53^ to remove any adaptor contamination, and Hi-C reads quality control checked using FastQC (v0.11.9)^49^. Genome profiling was performed using GenomeScope2 (v2.0)^54^ and a k-mer database for each species generated using Meryl (v1.3, *k* = 31)^55^. Phased haplotype contig-level assemblies were generated with Hifiasm (v0.19.0)^56^ using both HiFi and Hi-C reads. Sorted bam files containing alignment results of Hi-C reads to contig-level assemblies for each haplotype were produced following the Dovetail Genomics mapping pipeline^57^, and used to scaffold the assemblies with YAHS (v1.2a.2)^58^. Scaffold-level assemblies were screened for contaminant sequences (foreign organisms or mitochondrial) using both FCS-GX^59^ and Tiara (v1.0.3)^60^, and any contamination was removed. Hi-C contact maps were generated with PretextMap (v0.1.9) using Hi-C read alignments to decontaminated scaffold-level assemblies for each haplotype. Manual genome curation was undertaken using PretextView software (v0.2.5)^61^ to correct mis-assemblies, missed-assemblies, and to re-orient scaffolds. Quality assessment of final curated assemblies was performed using gfastats (v1.3.6) to generate summary statistics^62^, Benchmarking Universal Single-Copy Orthologs (BUSCO, v5.4.7) analysis using 3640 conserved single-copy Actinopterygii genes (actinopterygii_odb10) for gene content completeness^63^, and Merqury (v1.3) to assess base-level accuracy and completeness^55^. Blob plots and snail plots were generated using the Galaxy Australia implementation of BlobToolKit (Galaxy Version 4.0.7+galaxy2)^64,65^.

We performed a molecular validation of the nominal identity of our voucher specimens, samples and data, to provide supporting evidence that they represent the nominal species as opposed to cryptic diversity in the lineage, and as an internal quality control check to conform that tube or data swaps did not occur during sample processing. Complete mitochondrial genomes were assembled from the PacBio HiFi, Hi-C and RNA-Seq data that was generated for each specimen. Individual 12S and 16S ribosomal RNA, and Cytochrome Oxidase I (COI), sequences were mined from each mitogenome and queried against a custom internally curated database of 12S, 16S, CO1 and whole mitogenome sequences of marine vertebrates that were downloaded from NCBI Genbank and the Barcode of Life Data Systems (BOLD) database. Nominal identity was considered validated if high-confidence matches (>200 bp, >98% identity) against 12S, 16S and COI RefSeqs for the nominal species were returned. No reference data were available for *P. klunzingeri* at the time of study. In this case, we confirmed that identical 12S, 16S and CO1 sequences were retrieved from all the datasets we generated, and that the best matching RefSeqs in our database were congeneric (belonging to genus *Pempheris*).

Quality control checked HiFi and Hi-C sequence data and phased assemblies were uploaded together with the counterpart quality control checked RNA-Seq data for future genome annotation by NCBI according to their Eukaryotic Genome Annotation Pipeline^66^.

All code relating to genome assembly and analysis pipelines are accessible on GitHub: https://github.com/MinderooFoundation.

### Data descriptor

Characteristics and availability details for sequencing data input to the *E. armatus* (fEnoArm2) and *P. klunzingeri* (fPemKlu1) assemblies are presented in Table 2.

**Fig. 4:**
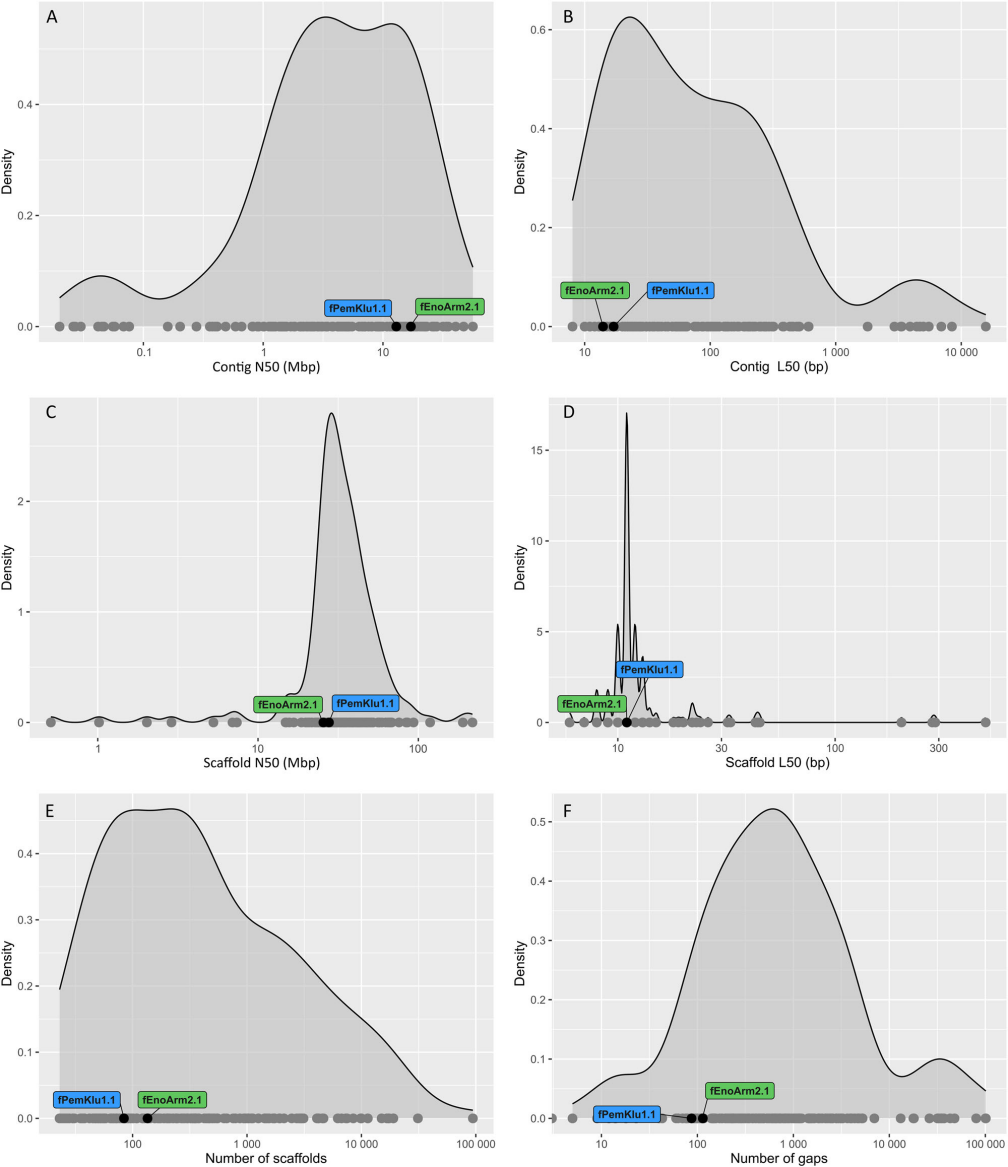
Comparison of genome assembly statistics for *Enoplosus armatus* and *Pempheris klunzingeri* and publicly available RefSeq genome assemblies. Grey dots represent publicly available genome assembly statistics (accessed from NCBI May 2024), and the black dots (*E. armatus* labelled green and *P. klunzingeri* labelled blue) show Ocean Genomes assemblies for **A** Contig N50 **B** Contig L50 **C** Scaffold N50 **D** Scaffold L50 **E** Number of scaffolds and **F** Number of gaps.

The *E. armatus* (fEnoArm2) and *P. klunzingeri* (fPemKlu1) assemblies are chromosome level (Supplementary Fig. 1) and satisfy the EBP version 6.0—September 2024 6.C.Q40^33^ and VGP-2020 7.c.P6.Q50.C95^9^ quality standards across all metrics (Table 3; Supplementary Fig. 2). Contiguity is very high, with an average of 4.1 assembly gaps per chromosome (Table 3). Both assemblies compare favourably to existing RefSeq^30^ assemblies for bony fish (Fig. 4).

**Table 3:** Assembly characteristics and data availability for *Enoplosus armatus* (fEnoArm2) *and Pempheris klunzingeri* (fPemKlu1) assemblies as compared to the EBP^33^ and VGP assembly standards^9^

	EBP v6.0 (6.C.Q40)	VGP-2020 (7.c.P6.Q50.C95)	*Enoplosus armatus* (fEnoArm2)		*Pempheris klunzingeri* (fPemKlu1)	
			Hap 1	Hap 2	Hap 1	Hap 2
NCBI BioProject			PRJNA1074348		PRJNA1079283	
Assembly size (Mb)			579.88	578.32	646.32	632.27
Number of scaffolds			135	94	442	84
Scaffold NG50 (Mb)	>10	=Chr. NG50	25.58	25.62	27.81	28.13
Contig NG50 (Mb)	>1	>10	17.10	15.96	13.65	14.88
BUSCO completeness (%)	>90	>95	98.9% [S:98.2%, D:0.7%]	99.0% [S:98.1%, D:0.9%]	99.3% [S:98.5%, D:0.8%]	99.3% [S:99.1%, D:0.2%]
Gaps	<1000 per Gb	<200 per Gb	114	100	87	93
Assembly assigned to chromosomes (%)	>90	>95	99.52	99.68	96.87	99.03
QV	>40	>50	61.18	60.18	57.65	62.38
			61.18		59.37	
Merqury completeness (%)	>90	>95	90.26	90.25	91.77	91.81
			99.76		99.73	

The *E. armatus* (fEnoArm2) assembly is almost entirely scaffolded on 2*n* = 48 chromosomes, with less than 0.5% of the assembly unplaced (Table 3; Supplementary Figs. 1 and 3). The assembled haplotypes of 580 Mb (Hap1) and 578 Mb (Hap2) are very close to the predicted haploid genome size of 579 Mb and each show very high completeness (>98.9% BUSCO, >99.7% Merqury) (Table 3).

The *P. klunzingeri* (fPemKlu1) haplotypes assembled at 646 Mb and 632 Mb, which is a little larger than the genome size of 591 Mb predicted during assembly, with over 96% (626 Mb) of each haplotype anchored to 2*n* = 48 chromosome scaffolds (Fig. 3; Supplementary Figs. 1 and 3). Overall, the completeness of the haplotype assemblies was very high (>99% by BUSCO and Merqury) (Table 3).

All sequencing data and genome assemblies produced by Ocean Genomes are accessible under NCBI BioProject number PRJNA1046164, and the affiliated SRA or GenBank records: https://www.ncbi.nlm.nih.gov/bioproject/1046164.

Sequence and assembly data for *E. armatus* and *P. klunzingeri* are accessible under NCBI accessions PRJNA1074348 and PRJNA1079283, respectively.

## Concluding remarks

The *E. armatus* (fEnoArm2) and *P. klunzingeri* (fPemKlu1) assemblies were produced by aligning with best practice protocols and quality standards proposed by global genome sequencing consortia. Our commitment to open data sharing ensures that these high-quality reference genome resources are freely available worldwide, fostering equitable access to benefits, collaboration and accelerating scientific progress in genomics-based studies of marine vertebrates. With a particular focus on high conservation value species and those native or endemic to Australian waters, Ocean Genomes intends to facilitate genomics-enabled biodiversity and conservation research on the marine vertebrate fauna from this region.

In these ways, Ocean Genomes is well-positioned to contribute valuable data for marine vertebrates toward the goal of sequencing representatives of all eukaryotic species under the EBP umbrella. While the species presented here represent ray-finned fishes, future releases of Ocean Genomes assemblies will be increasingly collaborative and encompass the diversity of marine vertebrates, including cartilaginous fishes, marine mammals, birds and reptiles, incorporating threatened and commercially important species.

## Supplementary information

Supplementary Information

## Data Availability

All Ocean Genomes sequencing data and genome assemblies will be openly accessible in the public domain and available for use under a Creative Commons Attributions license CC BY 4.0. A customised Minderoo OceanOmics dashboard provides regular updates to the community regarding collaborations, specimens acquired and prioritised for sequencing, the type of reference genome being produced and the progress of a sample from collection through to final assembly and data sharing. The dashboard connects users to open repositories (NCBI and Amazon Web Services (AWS)) where data and supporting resources are available for download (Figure 2). In future iterations of the dashboard, we intend to share standardised genome notes that promote the reuse of the data, and invite collaboration and disclosure of cultural authority and traditional knowledge interests of indigenous peoples and local communities, for example by incorporating biocultural, traditional knowledge and engagement notices (e.g. via institutional implementations of the CARE principles, or via the Local Contexts Notices system [https://localcontexts.org/](https://localcontexts.org) [ref. ^39^]. Ocean Genomes sequencing data and genome assemblies are also accessible directly via NCBI under BioProject number PRJNA1046164 and the affiliated Sequence Read Archive (SRA) or GenBank records. Progress toward high-quality reference genome production is also reported via Genomes on a Tree (GoaT) [40] as part of coordinated efforts across the EBP. All code relating to genome assembly and analysis pipelines are accessible on GitHub: https://github.com/MinderooFoundation. All sequencing data and genome assemblies produced by Ocean Genomes are accessible under NCBI BioProject number PRJNA1046164, and the affiliated Sequence Read Archive (SRA) or GenBank records: https://www.ncbi.nlm.nih.gov/bioproject/1046164.Sequence and assembly data for **Enoplosus armatus** and **Pempheris klunzingeri** are accessible under NCBI accessions PRJNA1074348 and PRJNA1079283, respectively.
