# Supplementary material for: Ocean Genomes: reference genome resources for marine vertebrates
Source: NPJ Biodivers. 2025 Oct 1;4:38. doi: 10.1038/s44185-025-00109-2 (PMC12488967; doi:10.1038/s44185-025-00109-2)
Supplement: Supplementary file 1 — Supplementary Information [file 44185_2025_109_MOESM1_ESM.pdf]

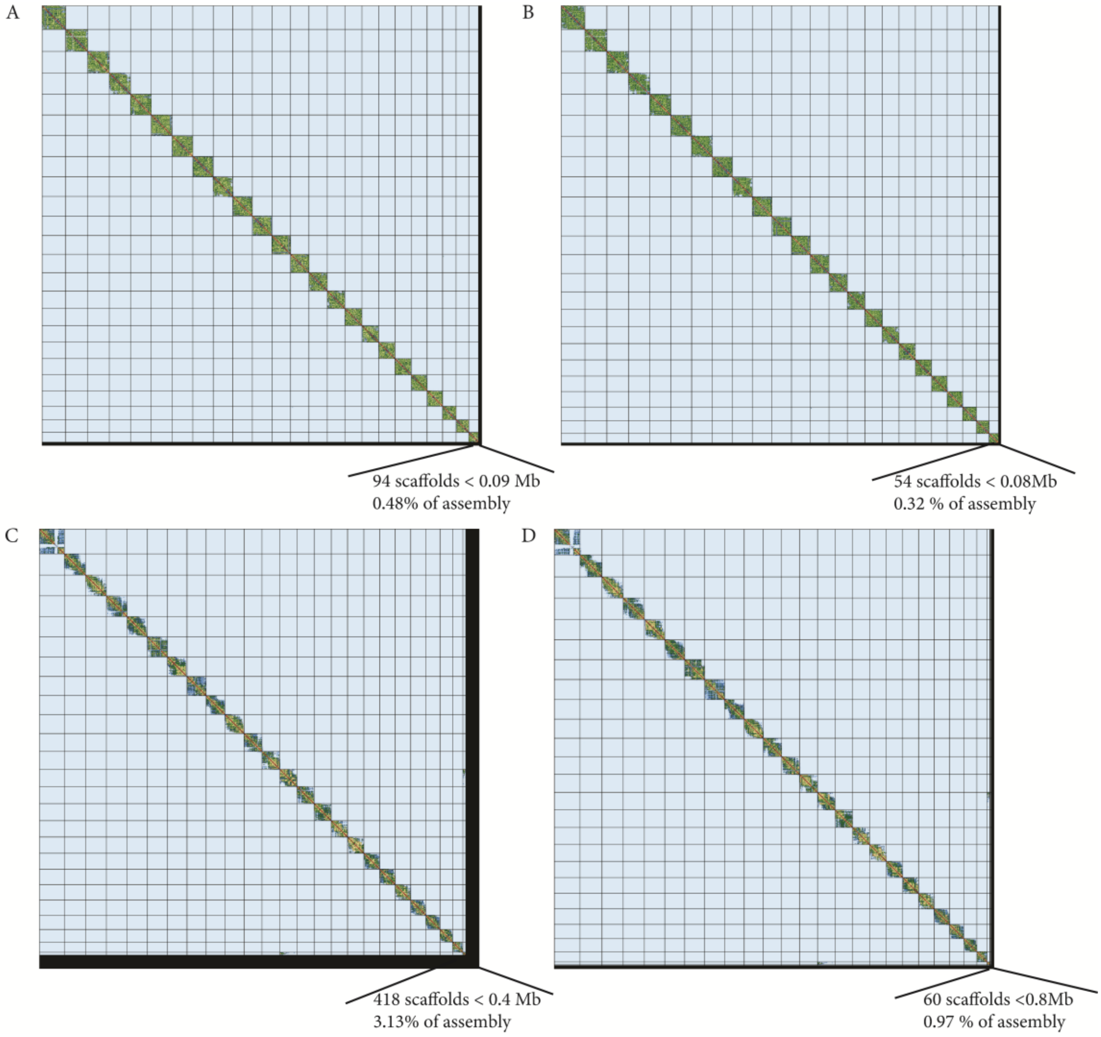

Supplementary Figure 1. Hi-C linked contact maps of the final curated genome assemblies of *Enoplosus armatus* a) haplotype 1, and b) haplotype 2; *Pempheris klunzingeri* c) haplotype 1, and d) haplotype 2. All chromosomes are ordered by length and the statistics detail how many scaffolds were not assigned to chromosomes, the maximum length of these scaffolds (Mb), followed by the percentage of the overall assembly they represent.

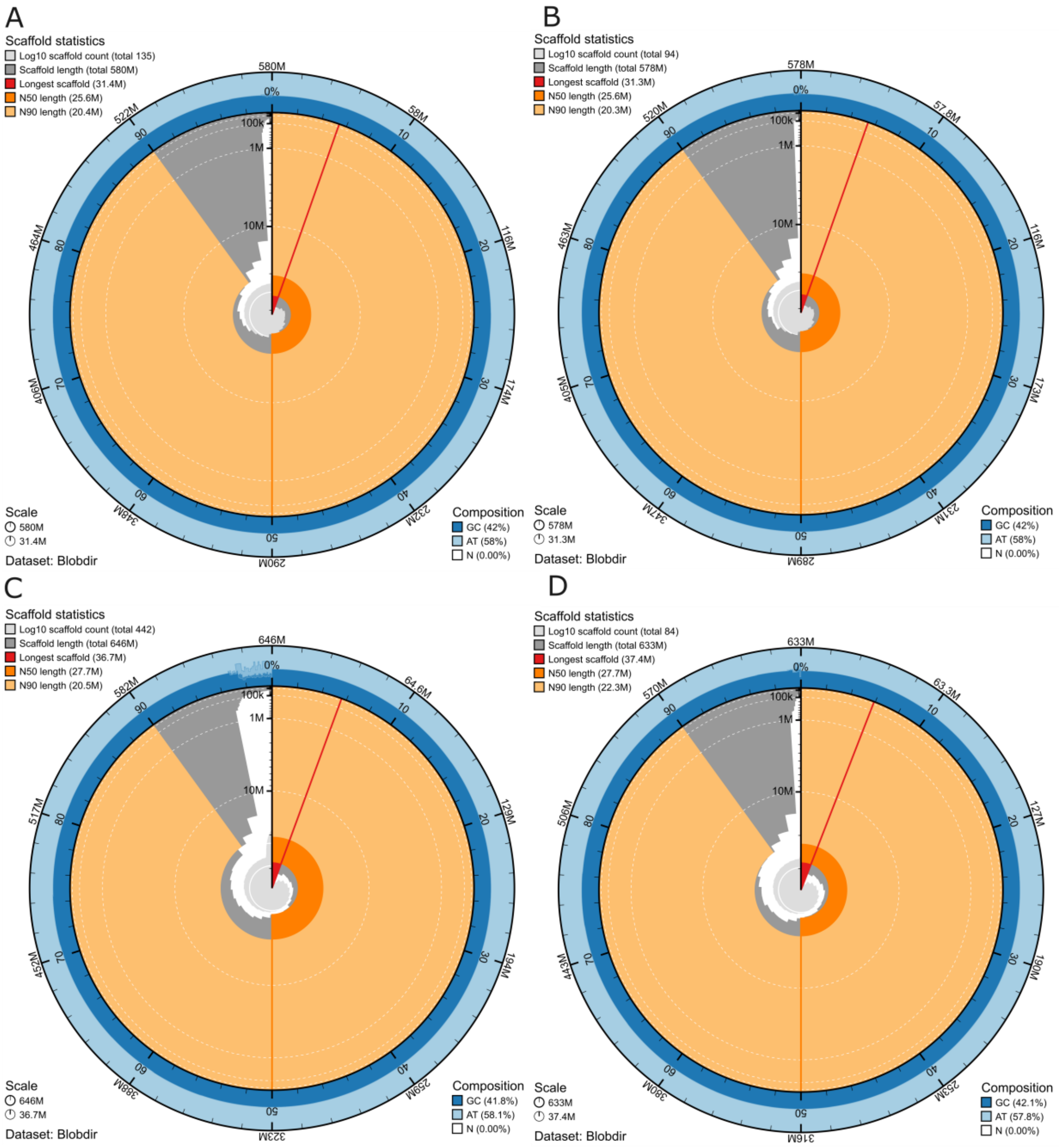

Supplementary Figure 2. Genome assembly metrics of the final curated genome assemblies of *Enoplosus armatus* a) haplotype 1, and b) haplotype 2; *Pempheris klunzingeri* c) haplotype 1, and d) haplotype 2. The BlobToolKit Snailplot shows N50 metrics and BUSCO gene completeness. The distribution of scaffold lengths is shown in dark grey with the plot radius scaled to the longest scaffold present in the assembly (shown in red). Orange and pale-orange arcs show the N50 and N90 scaffold lengths, respectively. The pale grey spiral shows the cumulative scaffold count on a log scale with white scale lines showing successive orders of magnitude. The blue and pale-blue area around the outside of the plot shows the distribution of GC, AT, and N percentages in the same bins as the inner plot.

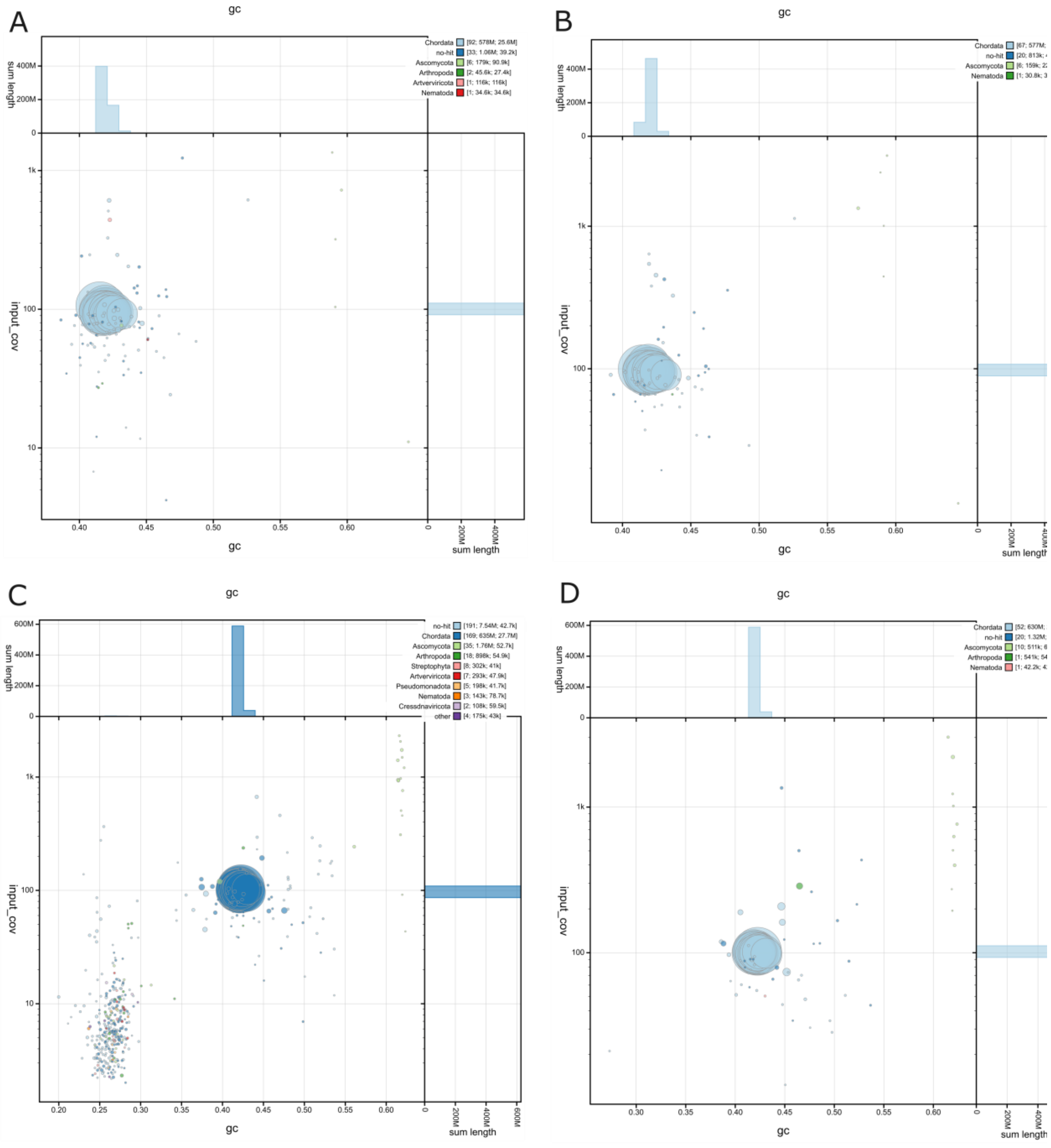

Supplementary Figure 3. Genome assembly coverage of the final curated genome assemblies of *Enoplosus armatus* a) haplotype 1, and b) haplotype 2; *Pempheris klunzingeri* c) haplotype 1, and d) haplotype 2. BlobToolKit GC-coverage plot. Scaffolds are coloured by phylum. Circles are sized in proportion to scaffold length. Histograms show the distribution of scaffold length sum along each axis.
